# D-limonene ameliorates metabolic dysfunction-associated steatotic liver disease by inhibiting the PPARγ/SCD-1 pathway and improving lipid metabolism disorders

**DOI:** 10.3389/fphar.2026.1843336

**Published:** 2026-07-02

**Authors:** Fang Wang, Yan Qing Liu, Jun Nan Wei, Xiao Fen Wang, Feng Qin Li, Xiu Jia Tian, Dan Li, Yu Xuan Gao, Xin Zhi Guo, Xue Yan Li, Ping Luo

**Affiliations:** 1 The Jiangxi Province Key Laboratory for Diagnosis, Treatment, and Rehabilitation of Cancer in Chinese Medicine, Mass Spectrometry Diagnosis and Treatment and Chronic Disease Rehabilitation Research Center, Jiangxi Engineering Research Center for Translational Cancer Technology, Jiangxi University of Chinese Medicine, Nanchang, China; 2 Teaching and Research Office of Chinese Medicine Resources, Jiangxi University of Chinese Medicine, Nanchang, China

**Keywords:** D-limonene, lipid metabolism, metabolic dysfunction-associated steatotic liver disease, multi-omics analysis, PPARγ, SCD-1

## Abstract

**Introduction:**

Metabolic dysfunction-associated steatotic liver disease (MASLD) is a highly prevalent chronic liver disorder characterized by intrahepatic lipid accumulation, oxidative stress, and inflammatory responses, underscoring the urgent need for effective therapeutic strategies. D-limonene, a cyclic monoterpenoid primarily derived from citrus fruits, possesses well-documented antioxidant, anti-fibrotic, and wound-healing properties. However, its therapeutic potential in MASLD remains largely unexplored.

**Methods:**

To evaluate the therapeutic efficacy of D-limonene, a rat model of MASLD was established using a high-fat diet (HFD). An integrated approach combining metabolomics, transcriptomics, and network analysis was employed to identify key signaling pathways and molecular targets. The expression levels of candidate targets were validated by RT-qPCR and Western blotting. Molecular docking simulations were performed to predict the binding affinity between D-limonene and the target proteins. Furthermore, a cellular thermal shift assay (CETSA) was conducted to confirm direct binding and protein stabilization. In parallel, cellular experiments using BRL-3A and HepG2 cells were carried out to assess the effect of D-limonene on lipid accumulation.

**Results:**

D-limonene treatment significantly ameliorated liver injury, reduced excessive lipid deposition, attenuated inflammation, and suppressed oxidative stress in HFD-induced rats. Integrated multi-omics and network analyses identified the PPAR signaling pathway as the core pathway mediating the regulatory effects of D-limonene on lipid metabolism, with PPARγ and SCD-1 pinpointed as key downstream targets. RT-qPCR and Western blot analyses confirmed that D-limonene significantly downregulated the expression of both PPARγ and SCD-1. Molecular docking simulations revealed stable binding of D-limonene to PPARγ and SCD-1. Importantly, CETSA further demonstrated that D-limonene directly binds to and stabilizes these two proteins. Consistently, cellular assays showed that D-limonene significantly reduced lipid accumulation in BRL-3A and HepG2 cells by inhibiting the PPARγ/SCD-1 axis.

**Conclusion:**

Collectively, these findings suggest that D-limonene alleviates HFD-induced MASLD, and its protective effects may be mediated, at least in part, through inhibition of the PPARγ/SCD-1 axis. This study provides preliminary evidence supporting the potential development of D-limonene as a targeted therapeutic agent for MASLD. Further studies are warranted to validate these findings and elucidate additional underlying mechanisms.

## 1 Introduction

Metabolic dysfunction-associated steatotic liver disease (MASLD) is a chronic hepatic disorder tightly linked to obesity and insulin resistance. The disease spectrum ranges from isolated hepatic steatosis to metabolic dysfunction-associated steatohepatitis (MASH) characterized by inflammation and hepatocellular injury, and may progress to fibrosis, cirrhosis, and ultimately hepatocellular carcinoma (HCC) (Chen et al., 2024). Moreover, with an estimated global prevalence of 25%–30% in adults, and exceeding 50% in high-risk populations such as type 2 diabetes patients (Younossi et al., 2023), MASLD has emerged as a leading cause of end-stage liver disease, imposing a substantial public-health burden. Despite recent advances, including the 2024 FDA approval of resmetirom (a thyroid hormone receptor-β agonist) (Harrison et al., 2024) and the hepatic benefits of glucagon-like peptide-1 (GLP-1) receptor agonists like semaglutide (Wang et al., 2025), effective treatment options are still limited. Therefore, the discovery of new therapeutics with well-characterized mechanisms and good safety profiles remains a critical unmet need.

The core pathological derangement in MASLD is hepatic lipid dysregulation, marked by increased lipogenesis, reduced fatty-acid oxidation, and diminished lipid export (Zhang et al., 2024). This imbalance leads to the accumulation of free fatty acids (FFA) and lipotoxic intermediates, which act not only as direct hepatotoxins but also as signaling molecules that induce mitochondrial dysfunction and endoplasmic reticulum stress. These events, in turn, fuel the generation of reactive oxygen species (ROS) and exacerbate oxidative damage (Fu et al., 2022; Li et al., 2021). Concurrently, the lipotoxic milieu activates Kupffer cells, promoting the release of pro-inflammatory cytokines such as tumor necrosis factor-alpha (TNF-α) and interleukin-6 (IL-6) and establishing a persistent, low-grade inflammatory state (Fan et al., 2023). Together, this self-reinforcing cycle of lipid overload, oxidative stress, and persistent inflammation drives disease progression from steatosis to MASH and ultimately to hepatic fibrosis (Steinberg et al., 2025).

Within this intricate pathological network, peroxisome proliferator-activated receptor γ (PPARγ) is widely recognized as a central pathological regulator. As a key nuclear transcription factor, PPARγ coordinates adipogenesis, lipid storage, and systemic insulin sensitivity. Its aberrant activation in the liver upregulates multiple lipogenic genes, directly promoting *de novo* lipogenesis and steatosis (Li et al., 2025). Mechanistically, PPARγ promotes hepatic lipid desaturation and anabolism by upregulating the rate-limiting enzyme stearoyl-CoA desaturase-1 (SCD-1), which converts saturated to monounsaturated fatty acids for triglyceride and phospholipid synthesis (Dillon et al., 2008). Moreover, catalytic products of SCD-1, such as oleic acid, can function as endogenous ligands that feedback to modulate PPARγ activity, amplifying lipogenic signaling and promoting MASLD development (Mori et al., 2024). Consequently, targeted disruption of the PPARγ/SCD-1 axis represents a promising therapeutic strategy to correct hepatic lipid dysregulation and halt disease progression.

Natural small molecules, especially those derived from the diet, constitute privileged source for modern drug discovery in metabolic disorders, owing to their polypharmacological properties and favorable safety profiles. D-Limonene, a monoterpene derived from citrus peel with Generally Recognized as Safe (GRAS) status, has recently emerged as a candidate of considerable translational potential. Mechanistically, D-limonene attenuates hepatic steatosis by synergistically activating PPARα and AMP-activated protein kinase (AMPK) signaling while concurrently suppressing LXR-β-mediated lipogenesis (Jing et al., 2013). Simultaneously, it exerts anti-inflammatory and antioxidant effects through the MAPK/Nrf2 and NF-κB pathways, thereby protecting hepatocytes from injury. Notably, in both carbon tetrachloride (CCl4)-induced and bile duct ligation models of hepatic fibrosis (Ahmad et al., 2018), D-limonene significantly reduces collagen deposition and α-SMA expression, conferring an anti-fibrotic advantage that is not presently achieved by resmetirom or GLP-1 receptor agonists. A recent randomized, placebo-controlled trial has further substantiated its clinical potential, demonstrating that D-limonene significantly reduces BMI and improves glycemic control in patients with MASLD, with a favorable tolerability profile (Tan et al., 2025). Collectively, by virtue of its multi-target modulation, excellent safety profile, and distinctive mechanisms of action, D-limonene holds considerable promise as an important addition to the therapeutic armamentarium for MASLD.

Despite these activities suggesting a potential role in modulating lipid metabolism, the therapeutic effect of D-limonene against MASLD and its underlying molecular mechanisms have not been fully elucidated. In this study, we systematically evaluated the impact of D-limonene on multiple MASLD-relevant outcomes and applied an integrated multi-omics approach to identify the key pathways and targets mediating its hepatoprotective effects, thereby providing a mechanistic basis for developing D-limonene as a candidate therapy for MASLD.

## 2 Materials and methods

### 2.1 Chemicals

D-limonene (Cat. No. D304599) and Orlistat (Cat. No.2409404A) were purchased from Shanghai Aladdin Biochemical Technology Co., Ltd. and Hangzhou Zhongmei Huadong Pharmaceutical Co., Ltd., respectively. Corn oil, HFD (D12451, 45% kcal from fat), and standard maintenance chow (D12450J) were obtained from Beijing Wanqian Jiaxing Biotechnology Co., Ltd. The pharmacological modulators T0070907 (Cat. No.HY-13202), A939572 (Cat. No.HY-50709), and rosiglitazone (Cat. No. 122320-73-4) were procured from MedChemExpress (South Brunswick, United States).

### 2.2 Network analysis

A network analysis approach was employed to investigate the potential targets and mechanisms of D-limonene against MASLD, following established protocols (Ye et al., 2025). Potential targets of D-limonene were predicted by integrating results from SwissTargetPrediction, Super-PRED, and PharmMapper; redundant entries were removed to compile a comprehensive drug-target set. Next, disease-associated genes were retrieved from GeneCards, OMIM, and DisGeNET using the search terms “non-alcoholic fatty liver disease” and “metabolic dysfunction-associated steatotic liver disease.” These results were merged and deduplicated to establish the MASLD-related gene pool. The intersection between the drug-target set and the disease-gene pool was then identified using Venny 2.1 to generate a candidate target list for D-limonene intervention in MASLD.

The intersecting targets were submitted to Metascape for Gene Ontology (GO) functional annotation and Kyoto Encyclopedia of Genes and Genomes (KEGG) pathway enrichment analysis, with a significance threshold of *P* < 0.05. In parallel, the shared target list was imported into STRING v12.0 to construct a protein-protein interaction (PPI) network, with a minimum interaction confidence score set >0.9. The resulting network was visualized and analyzed in Cytoscape 3.10.0, and the CytoHubba plug-in was applied to identify the top 10 hub genes based on the Degree algorithm, which were defined as core therapeutic targets.

### 2.3 Animal experiments

Forty male Sprague–Dawley rats (6 weeks old, 200 ± 20 g) were purchased from the Laboratory Animal Center of Jiangxi University of Chinese Medicine [license No. SCXK (Gan) 2018-0003]. All rats were housed under controlled conditions (22 °C ± 2 °C; 50% ± 10% humidity; 12/12-h light/dark cycle). All experimental procedures were approved by the Animal Ethics Committee of Jiangxi University of Traditional Chinese Medicine. Following a 7-day acclimatization period, the rats were randomly assigned to five groups (n = 8 per group): normal control, MASLD model, MASLD + orlistat (40 mg/kg) (Lim et al., 2012; Vijayalakshmi, 2014), MASLD + D-limonene-low (D-L, 100 mg/kg) (Sabbagh et al., 2025), and MASLD + D-limonene-high (D-H, 300 mg/kg) (Victor Antony Santiago et al., 2012).

To induce MASLD, all groups except the normal control were fed a HFD (45% fat, 35% carbohydrate, 20% protein) for 8 consecutive weeks (Qin et al., 2026), while the normal control group received standard maintenance chow throughout. After the induction period, a 3-week therapeutic intervention was conducted. During the intervention period, the normal control group and the MASLD model group received an equal volume of 0.9% normal saline. MASLD animals were treated with orlistat (40 mg/kg), D-limonene low dose (100 mg/kg), or D-limonene high dose (300 mg/kg), respectively.

At the end of the intervention period, rats were fasted for 12 h with free access to water. The following morning, fasting blood glucose (FBG) was measured using a portable glucometer via tail vein blood sampling. Subsequently, rats were anesthetized with ketamine and euthanized. Serum and liver tissues were collected and stored at −80 °C for further biochemical and histopathological analyses.

### 2.4 Serum biochemical and fasting blood glucose analysis

Serum biochemical parameters including triglycerides (TG; Cat. No. S03027), total cholesterol (TC; Cat. No. S03042), high-density lipoprotein (HDL; Cat. No. S03025), low-density lipoprotein (LDL; Cat. No. S03029), alanine aminotransferase (ALT; Cat. No. S03030), and aspartate aminotransferase (AST; Cat. No. S03040) were quantified using an automatic biochemical analyzer (Hitachi, Ltd., Kokubunji, Tokyo, Japan).

Hepatic oxidative stress markers, including superoxide dismutase (SOD; Cat. No. A001-3-2), myeloperoxidase (MPO; Cat. No. A044-1-1), malondialdehyde (MDA; Cat. No. A003-1-1), reduced glutathione (GSH; Cat. No. A006-2-1), glutathione peroxidase (GSH-Px; Cat. No. A005-1-1), as well as non-esterified fatty acids (NEFA; Cat. No. A042-2-1), were measured using commercial assay kits (Nanjing Jiancheng Bioengineering Institute, Nanjing, China).

Serum levels of the pro-inflammatory cytokines TNF-α (Cat. No. H052-1-2), IL-6 (Cat. No. H007-1-2), and IL-1β (Cat. No. H002-1-2) were measured with the corresponding enzyme-linked immunosorbent assay (ELISA) kits (Nanjing Jiancheng Bioengineering Institute) following the manufacturer’s instructions. Briefly, blank, standard, and sample wells were set up on 96-well plates. After incubation with the respective working solutions, the optical density was measured at 450 nm, and cytokine concentrations were calculated based on the standard curves.

### 2.5 Hematoxylin–eosin (H&E) and oil red O staining

For histological evaluation, liver samples were fixed in 4% paraformaldehyde for 24 h, followed by dehydration, paraffin embedding, and sectioning for H&E staining. To visualize neutral lipids, additional tissue specimens were immersed in 30% sucrose for 48 h, embedded in optimal cutting temperature (OCT) compound, and sectioned into 5-µm cryosections. After rinsing with 60% isopropanol, the cryosections were stained with Oil Red O following the manufacturer’s instructions. All stained sections were examined under a light microscope for histopathological assessment.

### 2.6 Transcriptomic analysis

Total RNA was extracted from liver tissues using TRIzol™ reagent. Sequencing libraries were subsequently prepared with the NEBNext® Ultra™ RNA Library Prep Kit for Illumina®. Briefly, RNA was fragmented to 200–300 bp, purified using AMPure XP magnetic beads, and reverse-transcribed into double-stranded cDNA with the SuperScript™ double-stranded cDNA synthesis kit and random hexamer primers. After end-repair, adapter ligation, enrichment, and purification, the final cDNA libraries were subjected to paired-end sequencing (150 bp) on an Illumina NovaSeq 6000 platform.

Differentially expressed genes (DEGs) were identified using DESeq2, with statistical significance defined as |log_2_ fold-change| ≥ 1 and an adjusted p-value (q-value) < 0.05. Functional annotation and pathway enrichment analysis were then conducted through GO and the KEGG database, using the hypergeometric test with a significance threshold of *P* < 0.05. All sequencing was performed by Hangzhou Lianchuan Biotechnology Co. Ltd.

### 2.7 Untargeted metabolomics

Metabolomic profiling was performed using liquid chromatography–tandem mass spectrometry (LC-MS/MS). Acquired data, including mass-to-charge ratio (m/z), retention time, and ion mode, were preprocessed prior to analysis. Multivariate statistical analysis was applied to examine overall sample distribution, with permutation testing conducted to prevent model overfitting. Features meeting the criteria of *P* < 0.05, |log_2_ fold-change| ≥ 1, and variable importance in projection (VIP) > 1 were defined as differential metabolites and visualized using volcano plots and heatmaps. Pathway enrichment analysis based on the KEGG database was carried out via the hypergeometric test (*P* < 0.05) to identify relevant metabolic pathways. Furthermore, functional interpretation was extended using FunMeta, a functional metabolomics platform, to construct metabolite–disease interaction networks and systematically infer the biological roles of key metabolites. All metabolomic analyses were conducted by Shanghai OE Biotech Co. Ltd.

### 2.8 Real-time quantitative polymerase chain reaction (RT-qPCR)

Total RNA was isolated from liver tissues with an RNA extraction kit (BiOG, Changzhou, China) and reverse-transcribed using a commercial reverse-transcription kit (Cat. No. 11141ES60). Quantitative PCR was performed using the resulting cDNA under the following cycling conditions: initial denaturation at 95 °C for 30 s, followed by 40 cycles of denaturation at 95 °C for 5 s and annealing/extension at 60 °C for 30 s. Gene expression levels were calculated using the 2^−ΔΔCT^ method. Primer sequences used in this study are provided in Supplementary Table S1.

### 2.9 Western blotting analysis

For liver tissue, approximately 50 mg of liver tissue was minced and homogenized in RIPA lysis buffer (Cat. No. G2002, Servicebio) using a tissue homogenizer. The lysate was centrifuged at 12,000 × g for 10 min at 4 °C, and the supernatant was collected for protein quantification using a BCA kit (Cat. No. HRX0119, Heruibio) assay.

For human hepatoma HepG2 cells, cells were seeded in 6-well culture plates and treated with 1 mM palmitic acid/oleic acid mixture for 24 h to induce steatosis. Subsequently, they were treated with D-limonene (3 μM), T0070907 (12 μM), A939572 (10 μM), rosiglitazone (100 μM), D-limonene + T0070907 (3 μM + 7 μM), D-limonene + A939572 (3 μM + 5 μM), or D-limonene + rosiglitazone (3 μM + 150 μM). After treatment, the cells were washed twice with ice-cold phosphate-buffered saline (PBS) and lysed in RIPA lysis buffer (Cat. No. G2002, Servicebio) supplemented with protease and phosphatase inhibitors. The lysates were then incubated on ice for 30 min, and centrifuged at 12,000 × g for 10 min at 4 °C. The supernatants were collected, and protein concentrations were determined using the BCA kit (Cat. No. HRX0119, Heruibio).

Protein samples were then mixed with loading buffer, denatured at 95 °C for 5 min, and separated by sodium dodecyl sulfate–polyacrylamide gel electrophoresis (SDS-PAGE). Subsequently, proteins were transferred onto polyvinylidene fluoride (PVDF) membranes using a wet transfer system.

After blocking with 5% non-fat milk for 2 h at 25 °C, the membranes were incubated overnight at 4 °C with the following primary antibodies: anti-PPARγ (1:5000, Cat. No. 16643-1-AP, Proteintech), anti-SCD-1 (1:2000, Cat. No. 28678-1-AP, Proteintech), anti-GAPDH (1:5000, Cat. No. GB15002-100, Servicebio), and anti-Tubulin (1:5000, Cat. No. GB15140-100, Servicebio). Following extensive washing, the membranes were incubated with horseradish peroxidase (HRP)-conjugated goat anti-rabbit secondary antibody (1:10,000, Cat. No. GB23204, Servicebio) for 1.5 h at 25 °C. Protein bands were detected using a chemiluminescence substrate and quantified with Image Lab software (version 4.0).

### 2.10 Molecular docking and molecular dynamic simulation

The 2D structure of D-limonene was retrieved from PubChem database (http://pubchem.ncbi.nlm.nih.gov/) and converted into a 3D conformation using ChemOffice. The receptor structure was obtained from the RCSB PDB (http://www.rcsb.org/), selecting the highest-resolution model available. Prior to docking, the protein was pre-processed in PyMOL by removing crystallographic water molecules and non-protein ligands (e.g., phosphate ions).

Docking simulations were performed with AutoDock Vina 1.5.6. In AutoDockTools, hydrogen atoms and charges were added to the receptor, while rotatable bonds were defined for the ligand. A grid box was set around the active site to confine the search. The resulting poses were ranked by binding free energy (ΔG, kcal/mol), and the lowest-energy conformation was selected as the optimal binding mode. Interaction diagrams and 3D binding poses were generated using Discovery Studio 2019 to visualize key hydrogen bonds and hydrophobic contacts.

### 2.11 Cell processing and staining methods

HepG2 and BRL-3A cells (obtained from Cell Bank of the Chinese Academy of Sciences, Shanghai, China) were cultured in MEM supplemented with 10% fetal bovine serum and 1% non-essential amino acids at 37 °C under 5% CO_2_. Upon reaching 70%–80% confluence, cells were detached with 0.25% trypsin and passaged. For viability assays, cells were seeded in 96-well plates, allowed to adhere for 24 h, and then treated with a palmitate/oleate mixture (1 mmol/L) for 24 to induce steatosis. After treatment with compounds at the indicated concentrations, 10 µL of CCK-8 working solution (prepared at a 1:9 ratio with complete medium) was added to each well and incubated for 30 min at 37 °C. Absorbance at 450 nm was measured using a microplate reader, and cell viability was calculated accordingly.

For cell staining, cells were seeded in 6-well culture plates and HepG2 cells treated with 1 mM palmitic acid/oleic acid mixture for 24 h to induce steatosis (Lee et al., 2019). Subsequently, they were treated with D-limonene (3 μM), T0070907 (12 μM) (Tripathy et al., 2014), A939572 (10 μM), rosiglitazone (100 μM) (Rondón-Ortiz et al., 2017), D-limonene + T0070907 (3 μM + 7 μM), D-limonene + A939572 (3 μM + 5 μM), or D-limonene + rosiglitazone (3 μM + 150 μM). BRL-3A cells were similarly treated with a mixture of 1 mmol/L palmitic acid and oleic acid for 24 h to induce cellular steatosis (Lee et al., 2019). Subsequently, they were treated with D-limonene (10 μM), T0070907 (5 μM), A939572 (6.25 μM), rosiglitazone (2.5 μM), D-limonene + T0070907 (5.5 μM + 2.5 μM), D-limonene + A939572 (5.5μM + 5 μM), or D-limonene + rosiglitazone (5.5 μM + 2.5 μM).

After treatment, the culture medium was aspirated, and the cells were gently washed twice with phosphate-buffered saline (PBS). The cells were then fixed with 4% paraformaldehyde for 20 min at room temperature, followed by two additional washes with PBS. The fixed cells were incubated with 60% isopropanol for 5 min to enhance permeability. A fresh Oil Red O working solution was prepared by mixing a 0.5% (w/v) stock solution of Oil Red O in isopropanol with distilled water at a ratio of 3:2, followed by filtration through a 0.22-μm filter to remove precipitates. The cells were stained with this working solution for 15 min at room temperature. After staining, the cells were washed three times with distilled water to remove unbound dye. Nuclei were counterstained with hematoxylin for 1–2 min, and then gently rinsed with tap water. Stained lipid droplets were visualized and imaged using an inverted light microscope.

### 2.12 Cellular thermal shift assay (CETSA)

The CESTA was carried out as previously reported (Chen et al., 2026; Su et al., 2026). After incubating HepG2 cells with or without 10 μM D-limonene for 24 h, cell lysates were collected and the protein concentration was adjusted to 2.0 mg/mL. The samples were then aliquoted into PCR tubes (50 μL/tube) and heated in a thermal cycler at a gradient of 37, 41, 45, 49, 53, 57, 61, and 65 °C for 3 min at each temperature. After heating, the samples were centrifuged at 20,000 g for 20 min at 4 °C to separate the soluble fraction (supernatant). Equal volumes of the supernatant were subjected to Western blot analysis as described in Section 2.9 to generate thermal melt curves.

### 2.13 Statistical analysis

All data were analyzed with GraphPad Prism 9.0. Two-group comparisons employed unpaired Student’s t-tests, while multiple-group comparisons used one-way *ANOVA* with Tukey’s *post hoc* test. Data are presented as mean ± SD. *P* < 0.05 was considered statistically significant.

## 3 Results

### 3.1 D-limonene ameliorates HFD-induced hepatic injury in MASLD rats

**FIGURE 1:**
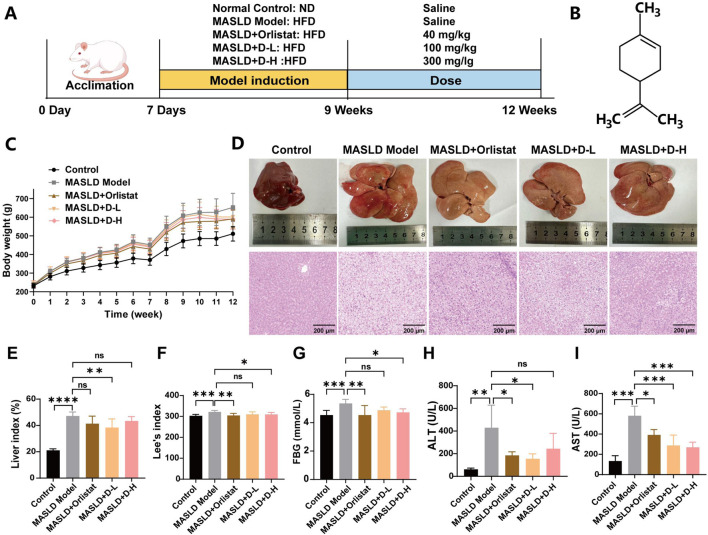
D-limonene attenuates HFD-induced hepatic injury in a MASLD rat model. **(A)** Schematic of the experimental timeline. **(B)** Chemical structure of D-limonene. **(C)** Body weight of rats in each group. **(D)** Representative photographs of gross liver morphology (top) and corresponding H&E-stained sections (bottom). **(E)** Hepatic index, calculated as (liver weight/body weight) × 100%. **(F)** Lee’s index, determined by [body weight (g)^(1/3)^ × 10/body length (cm)]. **(G)** Fasting Blood Glucose. **(H,I)** Serum levels of ALT and AST levels, measured using an automatic biochemical analyzer. *P < 0.05, **P < 0.01, ***P < 0.001, ****P < 0.0001, and ns: not significant. MASLD + Orlistat: Orlistat at 40 mg/kg; MASLD + D–L: D-limonene at 100 mg/kg; MASLD + D–H: D-limonene at 300 mg/kg.

To evaluate the therapeutic potential of D-limonene against MASLD, an HFD-induced rat model was employed (Figure 1A). The chemical structure of D-limonene is shown in Figure 1B. At the end of the intervention period (10 weeks), rats in the MASLD model group exhibited significant increases in body weight, liver index, and Lee’s index compared with normal controls (Figures 1C–F). Treatment of MASLD rats with orlistat (40 mg/kg) or D-limonene (100 and 300 mg/kg) attenuated these increases to varying degrees (Figures 1C–F). Similarly, FBG levels in the treatment groups were lower than those in the MASLD model group, although the most pronounced effects were observed in the MASLD + orlistat and MASLD + D-H groups (Figure 1G). Macroscopically, livers from normal rats appeared dark-red and smooth, whereas those from HFD-fed rats were pale and granular, indicating substantial lipid accumulation (Figure 1D). These pathological changes were notably ameliorated following administration of orlistat or D-limonene (Figure 1D).

H&E staining further demonstrated extensive intracellular lipid vacuolation and hepatocyte ballooning in the MASLD model group, confirming pronounced steatosis and hepatocellular injury (Figure 1D). These histopathological changes were dose-dependently mitigated by D-limonene treatment (Figure 1D). Consistently, serum ALT and AST activities were significantly elevated in HFD-fed rats, reflecting hepatic damage (Figures 1H,I). Both orlistat and D-limonene markedly reduced ALT and AST levels, further supporting their hepatoprotective efficacy (Figures 1H,I). Together, these results indicate that D-limonene effectively attenuates HFD-induced hepatic steatosis and functional impairment, highlighting its potential as a therapeutic agent for MASLD.

### 3.2 D-limonene reduces lipid deposition and circulating lipid levels

**FIGURE 2:**
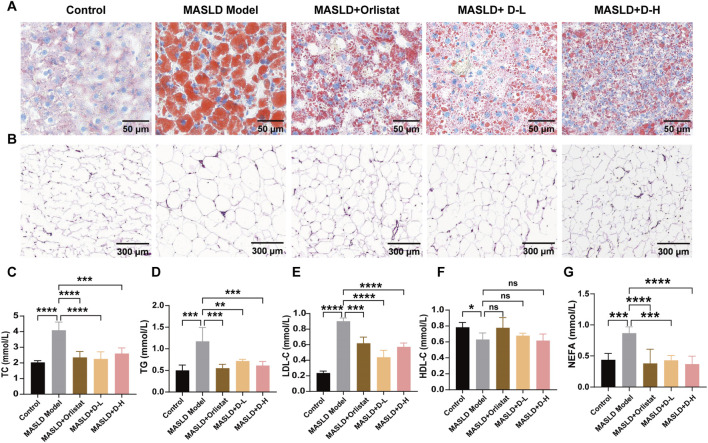
D-limonene reduces hepatic and adipose lipid deposition and improves serum lipid profiles. **(A)** Representative Oil Red O-stained sections of liver. **(B)** Representative H&E-stained sections of epididymal adipose tissue. **(C–F)** Serum levels of TG, TC, LDL-C, and HDL-C measured with an automatic biochemical analyzer (n = 6). **(G)** Serum NEFA concentration determined using a commercial assay kit (n = 6). *P < 0.05, **P < 0.01, ***P < 0.001, ****P < 0.0001, and ns: not significant. MASLD + Orlistat: Orlistat at 40 mg/kg; MASLD + D–L: D-limonene at 100 mg/kg; MASLD + D–H: D-limonene at 300 mg/kg.

To evaluate the effect of D-limonene on lipid deposition in the liver and adipose tissue, the researchers performed Oil Red O staining on liver sections and H&E staining on adipose tissue sections, respectively (Yang et al., 2025). Compared with the normal group, the model group exhibited extensive lipid droplet accumulation and hepatocyte enlargement, consistent with severe hepatic steatosis (Figure 2A) and epididymal adipose expansion (Figure 2B). Treatment with orlistat or D-limonene at either dose significantly reduced the abundance of lipids droplets in both liver and adipose tissue, along with a decrease in adipocyte size (Figures 2A,B). These data suggest that D-limonene effectively reverses HFD-induced excessive lipogenesis.

To further validate these findings, serum lipid profiles were analyzed. High-fat feeding significantly increased TG, TC, and LDL-C, while decreasing HDL-C compared with normal controls (Figures 2C–F), confirming the development of dyslipidemia. Treatment with orlistat or D-limonene restored TG, TC, and LDL-C to near-normal levels (Figures 2C–F). Given that elevated NEFA are a key driver of hepatic lipid accumulation, insulin resistance, and inflammation (Li et al., 2026), serum FFA were also measured. HFD-fed rats exhibited markedly higher NEFA levels, which were significantly reduced by both orlistat and D-limonene (Figure 2G). Collectively, these findings suggest that D-limonene alleviates lipotoxicity and related metabolic disturbances by reducing lipid deposition in liver and adipose tissue, while also lowering circulating levels of pathogenic lipids including TG, TC, LDL-C, and NEFA.

### 3.3 D-limonene ameliorates hepatic injury by suppressing hepatic inflammation and oxidative stress

**FIGURE 3:**
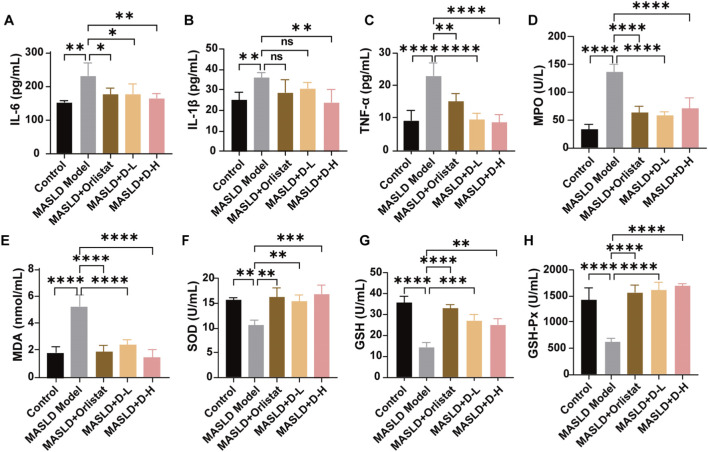
D-limonene alleviates hepatic inflammation and oxidative stress. **(A–C)** Serum IL-6, IL-1β, and TNF-α levels were measured by ELISA (n = 6). **(D–H)** Serum activities of MPO, MDA, SOD, GSH, and GSH-Px were determined using commercial assay kits (n = 4). *P < 0.05, **P < 0.01, ***P < 0.001, ****P < 0.0001, and ns: not significant. MASLD + Orlistat: Orlistat at 40 mg/kg; MASLD + D–L: MASLD + D-limonene at 100 mg/kg; D–H: D-limonene at 300 mg/kg.

Inflammation and oxidative stress are established as central drivers in the pathogenesis of MASLD, activating intrahepatic stress pathways, accelerating lipid peroxidation, promoting immune cell infiltration, and exacerbating insulin resistance—thereby advancing disease progression from simple steatosis to steatohepatitis and fibrosis (Fang et al., 2024). Compared with normal controls, the model group showed significant elevations in serum levels of IL-6, IL-1β, and TNF-α (Figures 3A–C), indicating a heightened inflammatory state. Treatment with orlistat or D-limonene substantially reduced these cytokine levels (Figures 3A–C), demonstrating the potent anti-inflammatory effect of D-limonene in the context of MASLD.

As pivotal indicators of oxidative status, MPO and MDA were measured as key markers of lipid peroxidation and oxidative tissue damage, while SOD, GSH and GSH-Px were assessed to evaluate endogenous antioxidant defense (Malyar et al., 2020). In the present model, MASLD rats displayed a pronounced oxidative imbalance compared with normal controls. This was evidenced by elevated serum MPO activity and MDA content (Figures 3D,E), alongside reduced activities of SOD, reduced GSH, and GSH-Px (Figures 3F–H), collectively indicating a compromised antioxidant defense and heightened oxidative stress. Treatment with orlistat or D-limonene effectively reversed these alterations, restoring all measured indices to near-normal levels (Figures 3D–H). Together, these results suggest that D-limonene enhances endogenous antioxidant capacity and reduces lipid-peroxidation-mediated damage, thereby contributing to hepatic protection in MASLD.

### 3.4 The PPAR pathway serves as the candidate mechanism underlying D-limonene’s protection of MASLD

**FIGURE 4:**
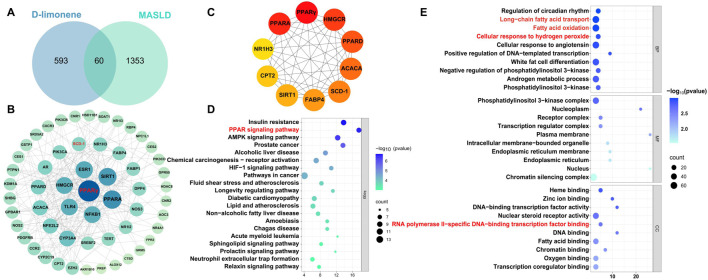
Network analysis suggests that PPAR signaling is potentially associated with the improvement of MASLD by D-limonene. **(A)** Venn diagram showing the intersection of D-limonene targets and MASLD-related genes. **(B)** PPI network of the overlapping targets. **(C)** Core PPI subnetwork identified by CytoHubba. **(D)** Bubble plot of KEGG pathway enrichment for the intersecting targets. **(E)** Bar chart of GO functional enrichment for the intersecting targets.

To elucidate the potential targets and mechanisms through which D-limonene alleviates MASLD, a network analysis approach was employed. Target prediction using PharmMapper, SwissTargetPrediction, and Super-PRED databases identified 1,413 non-redundant potential targets of D-limonene. Meanwhile, 653 MASLD-related genes were compiled from GeneCards, OMIM, and DisGeNET. Intersection of these two datasets revealed 60 shared targets (Figure 4A). “D-limonene–MASLD” interaction network consisting of 60 nodes and 269 edges was constructed in Cytoscape (Figure 4B). Topological analysis using the CytoHubba plug-in identified the top 10 hub genes, ranked as follows: PPARγ, PPARα, HMGCR, PPARD, ACACA, FABP4, SCD-1, SIRT1, CPT2, and NR1H3 (Figure 4C).

KEGG pathway enrichment analysis of the 60 intersecting targets identified 132 significantly enriched pathways. As shown in Figure 4D, the top 16 pathways, including insulin resistance, PPAR signaling, AMPK signaling, and prolactin signaling, are closely associated with metabolism and disease. These pathways are well-established regulators of metabolic disorders and tumorigenesis (Carling, 2017; Michalik et al., 2006; Samuel and Shulman, 2012), suggesting that these signaling networks may be involved in the effects of D-limonene on MASLD, providing a hypothesis for future experimental validation.

We next performed GO enrichment analysis on the 60 intersecting targets. Within the biological-process domain, the genes were significantly enriched for “long-chain fatty acid transport”, “fatty acid oxidation” and “cellular response to hydrogen peroxide” (Figure 4E), implying that D-limonene treatment is associated with pathways related to oxidative stress and lipid homeostasis. Molecular-function analysis localized these targets to key sites such as the endoplasmic-reticulum membrane, nucleus, and plasma membrane (Figure 4E), which are central to protein synthesis, lipid metabolism, and signal transduction. Cellular-component terms highlighted both “fatty acid binding” and “RNA polymerase II transcription factor binding” (Figure 4E), which correspond precisely to the canonical functions of a ligand-activated nuclear receptor such as PPARγ (Li et al., 2025). This suggests that D-limonene may modulate PPARγ activity to regulate downstream targets. In summary, the PPAR signaling axis is the most highly enriched pathway in our network analysis, and PPARγ is among the top-ranked hub genes. These results suggest that PPAR signaling may play an important role in the response to D-limonene in the context of MASLD, but this hypothesis requires direct experimental testing.

### 3.5 D-limonene reprograms PPAR-driven metabolism

**FIGURE 5:**
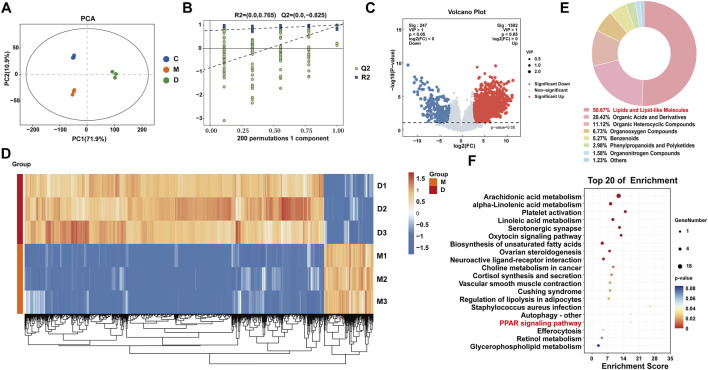
D-limonene reprograms hepatic metabolites linked to the PPAR pathway. **(A)** PCA score plot showing metabolic profiles of control, model, and high-dose D-limonene-treated groups. **(B)** The permutations test of metabolomics of control, model, and high-dose D-limonene-treated groups. **(C)** Volcano plot displaying DEMs between model and D-limonene-treated groups. **(D)** Heatmap visualizing DEMs in model versus D-limonene-treated groups. **(E)** Pie chart showing metabolite distribution between model and D-limonene-treated groups. **(F)** KEGG enrichment analysis of DEMs between model and D-limonene-treated groups.

To further investigate the mechanism, we conducted untargeted metabolomic profiling of liver tissues. Principal component analysis (PCA) of the metabolic data revealed distinct separations among the normal control, model, and high-dose D-limonene-treated groups in the score plot (Figure 5A), and the analysis model wasreliable after 200 permutation tests (Figure 5B). These results suggest that D-limonene substantially reshapes the liver metabolome in MASLD rats.

By comparing the model and D-limonene-treated groups in the OPLS-DA model and applying thresholds of variable importance in projection (VIP) ≥ 1.0 and |fold change| ≥ 2, we identified 1,749 differentially expressed metabolites (DEMs), consisting of 1,502 upregulated and 247 downregulated metabolites (Figures 5C,D). Among these, 50.67% of the differential metabolites were closely linked to lipid metabolism processes (Figure 5E). These DEMs were significantly enriched in 92 KEGG pathways (FDR <0.05), 47 of which were directly related to metabolic processes, including arachidonic acid, α-linolenic acid, and linoleic acid metabolism, and PPAR signaling (Figure 5F). Together, these findings indicate that D-limonene markedly reprograms the hepatic metabolome in MASLD rats, likely through regulation of key metabolic pathways, notably the PPAR axis, thus corroborating network analysis predictions and revealing a cohesive mechanism for its therapeutic effect.

### 3.6 D-limonene regulates the expression of PPAR-pathway-related genes

**FIGURE 6:**
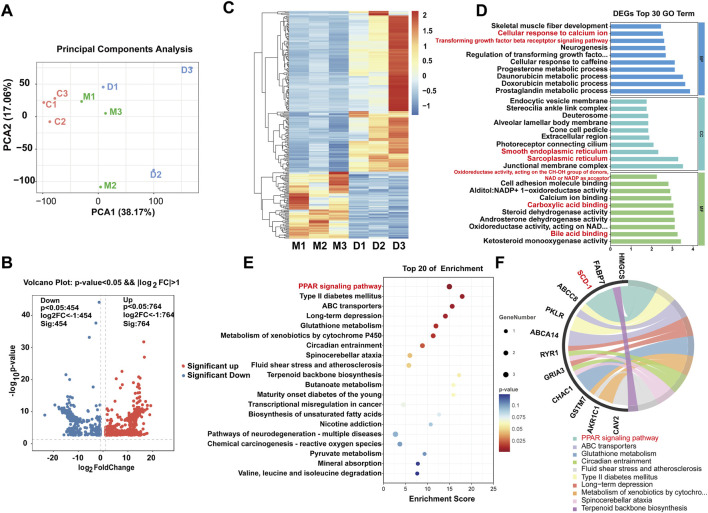
Transcriptomic analysis suggests D-limonene modulates gene expression linked to the PPAR pathway. **(A)** PCA score plot of control, model, and high-dose D-limonene-treated groups. **(B)** Volcano plot of DEGs between model and D-limonene-treated groups. **(C)** Heatmap of DEGs between model and D-limonene-treated groups. **(D)** GO enrichment analysis of DEGs between model and D-limonene-treated groups. **(E,F)** KEGG pathway enrichment analysis of DEGs between model and D-limonene-treated groups.

To validate the predictions from network analysis and further delineate the downstream transcriptional changes suggested by metabolomics, we performed RNA sequencing on liver tissues. PCA showed clear separation among the control, model, and high-dose D-limonene-treated groups (Figure 6A), reflecting distinct global transcriptional profiles. Using thresholds of |fold change| ≥ 2 and false discovery rate (FDR) < 0.05, we identified 1,218 differentially expressed genes (DEGs), including 764 upregulated and 454 downregulated transcripts (Figure 6B). Hierarchical clustering analysis further revealed that the expression pattern in D-limonene-treated groups was largely reversed compared with the model group (Figure 6C).

GO enrichment analysis of the DEGs was subsequently conducted to further define the biological functions modulated by D-limonene. At the biological-process level, the DEGs were notably enriched in “cellular response to calcium ion” and the “transforming growth factor-β (TGF-β) receptor signaling pathway” (Figure 6D). At the cellular-component level, terms such as “smooth endoplasmic reticulum” and “sarcoplasmic reticulum” (Figure 6D) were enriched, indicating a potential role of D-limonene in regulating lipid synthesis through the maintenance of endoplasmic-reticulum integrity and calcium homeostasis. Molecular-function analysis further highlighted terms including “oxidoreductase activity,” “carboxylic acid binding,” and “bile acid binding” (Figure 6D). These findings suggest that D-limonene may alleviate lipotoxic injury in MASLD by enhancing hepatic antioxidant capacity and regulating bile acid metabolism, consistent with the biochemical and inflammatory data presented earlier (Figure 3).

KEGG pathway analysis further demonstrated that the PPAR signaling pathway, type II diabetes mellitus, and related metabolic cascades were significantly enriched, with the PPAR axis showing the most prominent enrichment (FDR <0.001) (Figure 6E). Considering the established role of PPAR signaling in regulating fatty-acid oxidation, lipid transport, and energy homeostasis, together with its consistent identification in our network-pharmacology and metabolomics analyses, we next focused on the expression of PPAR-pathway-associated genes. As shown in Figure 6F, key genes such as SCD-1, FABP7, and HMGCS1 were markedly enriched within the PPAR pathway. Moreover, the observed change in SCD-1 expression was consistent with network-pharmacology predictions, supporting the need for subsequent experimental validation.

### 3.7 Integrated multi-omics analysis suggests D-limonene modulates hepatic lipid remodeling through the PPARγ/SCD-1 axis

**FIGURE 7:**
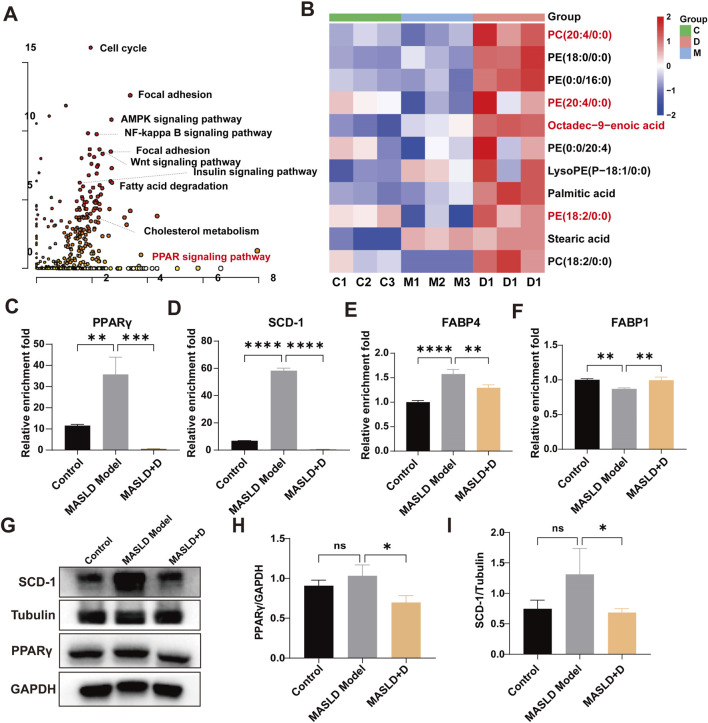
Integrated multi-omics analysis validates the PPARγ/SCD-1 axis as a key target of D-limonene. **(A)** Correlation analysis between DEGs and DEMs, presented as a scatter plot. **(B)** Metabolite heatmap of the control, model, and high-dose D-limonene treated groups. **(C–F)** mRNA expression levels of PPARγ, SCD-1, FABP4, and FABP1 determined by RT-qPCR. **(G–I)** Protein levels of PPARγ and SCD-1 assessed by Western blot. *P < 0.05, **P < 0.01, ***P < 0.001, ****P < 0.0001, and ns: not significant.

To clarify the regulatory interplay between transcriptional and metabolic changes, we performed integrated pathway analysis of DEGs and DEMs using Impala and MetaboAnalyst. KEGG enrichment consistently highlighted “PPAR signaling”, “AMPK signaling”, “insulin signaling” and “fatty acid degradation” as significantly co-enriched pathways (Figure 7A), suggesting that modulation of lipid metabolism and energy homeostasis is central to D-limonene’s mechanism of action.

Metabolomic profiling revealed pronounced phospholipid remodeling in response to D-limonene treatment. Notably, saturated lysophosphatidylethanolamine species—PE (18:0/0:0) and PE (0:0/16:0), which are enriched with fatty acid moieties that serve as primary substrates for SCD-1-mediated desaturation—were significantly accumulated in the D-limonene group compared to the model group (Figure 7B). Concurrently, polyunsaturated lysophospholipid—including PE (18:2/0:0), PE (20:4/0:0), and PC(20:4/0:0)—were significantly upregulated in the D-limonene group compared with both the model and control groups (Figure 7B), suggesting that D-limonene may drive phospholipid profile remodeling toward a polyunsaturated state by promoting the incorporation of polyunsaturated fatty acids into membrane phospholipids. Interestingly, oleic acid (Octadec-9-enoic acid) remained elevated in the D-limonene group despite SCD-1 suppression. This observation suggests enhanced lipid droplet mobilization, a process consistent with PPARγ inhibition, coupled with the redirection of fatty acid flux toward membrane phospholipid remodeling (Figure 7B). This shift from a monounsaturated fatty acid (MUFA)-rich phospholipid profile in MASLD toward a more balanced polyunsaturated state underscores D-limonene’s role in hepatic lipid remodeling.

Based on these multi-omics findings, we subsequently examined expression changes across PPAR family isoforms to identify key targets mediating D-limonene effects. RT-qPCR results demonstrated that PPARγ, SCD-1, and FABP4 mRNA levels were significantly upregulated in the model group compared with controls, and D-limonene treatment markedly suppressed the expression of these genes (Figures 7C–E). Conversely, the expression of FABP1, essential for hepatic fatty acid transport and typically reduced in MASLD (Guzmán et al., 2013), was restored following intervention (Figure 7F).

To further clarify the specific PPAR isoform targeted by D-limonene, we also evaluated PPARα and PPARβ (Supplementary Figure S1). Interestingly, both were upregulated in the MASLD model and suppressed following intervention. Given that conventional pharmacological activation of PPARα/β typically promotes lipid clearance (Mastoridou et al., 2026), their paradoxical elevation in the model group likely reflects a compensatory metabolic response to lipid overload. The fact that D-limonene decreased rather than enhanced their expression indirectly suggests that PPARα and PPARβ are not the primary therapeutic targets. Instead, the expression pattern of PPARγ, a core driver of lipogenesis, perfectly parallels the disease progression and therapeutic outcomes. Therefore, we selected the PPARγ/SCD-1 axis as the principal mechanism for in-depth investigation.

To validate these alterations at the translational level, Western blot analysis was performed. The results revealed significantly elevated hepatic PPARγ and SCD-1 protein levels in the MASLD model, which were substantially reduced following D-limonene intervention (Figures 7G–I). Collectively, these data suggest that D-limonene alleviates MASLD by suppressing PPARγ and its downstream target SCD-1, thereby re-balancing lipid metabolism.

### 3.8 Molecular dynamics simulation displays stable binding of D-limonene to PPARγ and SCD-1

**FIGURE 8:**
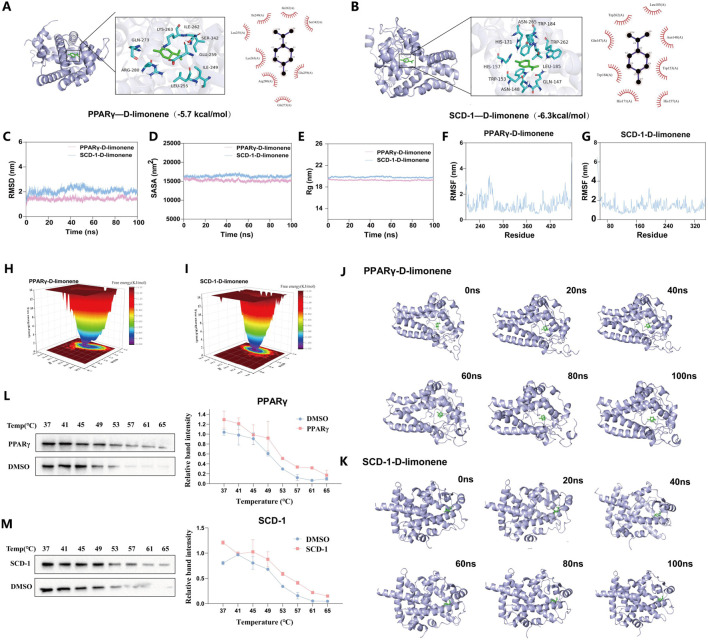
Molecular dynamics simulations validate stable binding of D-limonene to PPARγ and SCD-1. **(A,B)** Three-dimensional and two-dimensional representations of the docking poses of D-limonene with PPARγ **(A)** and SCD-1 **(B)**. **(C–G)** Time-dependent evolution of RMSD, SASA, Rg, and RMSF during the simulations. **(H,I)** Three-dimensional free-energy landscapes projected onto RMSD and Rg. **(J,K)** Dynamic evolution of the interaction between D-limonene and PPARγ **(J)** or SCD-1 **(K)** throughout the 100-ns simulation. **(L,M)** Binding affinity of D-limonene with PPARγ and SCD-1. **(L)** CETSA results for D-limonene binding to PPARγ. **(M)** Quantitative CETSA results for D-limonene binding to SCD-1.

To characterize the binding properties and conformational stability of D-limonene with the core targets PPARγ and SCD-1, molecular dynamics simulations were performed. Molecular docking calculations revealed that D-limonene forms stable complexes with both PPARγ and SCD-1, with favorable binding energies of −5.7 kcal/mol (Figure 8A) and −6.3 kcal/mol (Figure 8B), respectively, indicating a strong affinity for both proteins.

During the final 60–80 ns, the backbone root mean square deviation (RMSD) of both PPARγ and SCD-1 exhibited relative stabilization (Figure 8C), indicating minimal overall structural fluctuations. Consistent solvent accessible surface area (SASA) and radius of gyration (Rg) profiles further suggested no significant expansion or compression of the proteins (Figures 8D,E). Both complexes showed low residue fluctuations (Root-mean-square fluctuation, RMSF <3 Å), reflecting enhanced rigidity upon ligand binding (Figures 8F,G). Free-energy landscapes confined to low-energy basins indicated thermodynamically stable conformations (Figures 8H,I). Trajectory analysis revealed that D-limonene remained stably bound within both pockets (Figures 8J,K). Specifically, the D-limonene–PPARγ complex equilibrated rapidly (∼5 ns) and fluctuated around 1.5 Å, while the D-limonene–SCD-1 complex stabilized later (∼90 ns) at ∼2.2 Å, indicating that PPARγ exhibits greater conformational rigidity.

To experimentally validate whether D-limonene directly engages these targets, CETSA confirmed that D-limonene directly bound to and stabilized the PPARγ and SCD-1 proteins, demonstrating that these two molecules are genuine cellular targets of D-limonene (Figures 8L,M). Overall, these results suggest that D-limonene stably occupies the catalytic sites of PPARγ and SCD-1, suppressing their activities and thereby contributing to the amelioration of MASLD.

### 3.9 D-limonene reduces lipid accumulation through the PPARγ/SCD-1 axis

**FIGURE 9:**
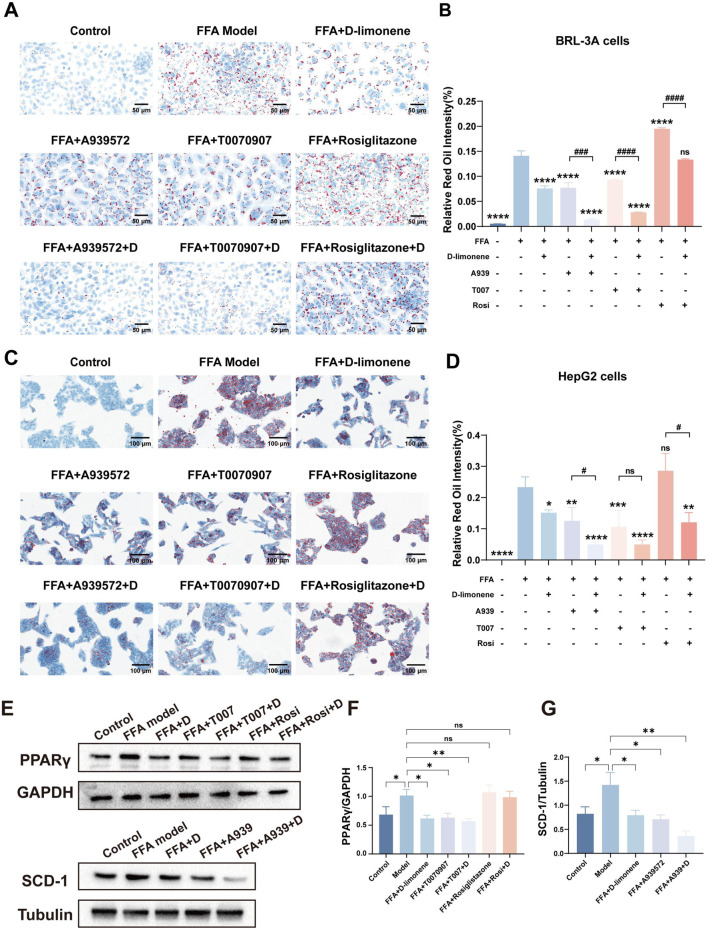
D-limonene reduces lipid accumulation in hepatocytes by targeting the PPARγ/SCD-1 axis. **(A)** Representative Oil Red O-stained images of rat BRL-3A hepatocytes treated as indicated (scale bar = 50 μm). **(B)** Quantification of lipid-droplet area in BRL-3A cells (n = 3). **(C)** Representative Oil Red O-stained images of human HepG2 hepatocytes under different treatments (scale bar = 100 μm). **(D)** Quantification of lipid-droplet area in HepG2 cells (n = 3). **(E–G)** Protein levels of PPARγ and SCD-1 assessed by Western blot. Compared with the FFA model group, *P < 0.05, ***P < 0.001, ****P < 0.0001, and ns: not significant; ^#^ *P* < 0.05, ^###^ *P* < 0.001, ^####^ *P* < 0.0001 (A939: A939572, SCD-1antagonist; T007: T0070907, PPARγ antagonist; Rosi: rosiglitazone, PPARγ agonist). See Section 2.11 for detailed concentrations.

To examine whether D-limonene modulates hepatocyte lipid metabolism via the PPARγ/SCD-1 axis, BRL-3A and HepG2 cells were treated with a FFA mixture (oleate: palmitate = 1:2) to induce a cellular model of lipid accumulation. Oil Red O staining showed substantial lipid-droplet accumulation after FFA exposure, which was markedly attenuated by D-limonene treatment (Figures 9A–D). These cellular findings are consistent with the histological improvements observed *in vivo* and confirm the lipid-lowering effect of D-limonene at the cellular level.

To functionally validate the PPARγ/SCD-1 axis as the primary target of D-limonene, we employed selective pharmacological agents. As shown in Figures 9A,C, the PPARγ antagonist T0070907 or the SCD-1 inhibitor A939572 alone significantly reduced lipid accumulation, while the PPARγ agonist rosiglitazone aggravated steatosis. When combined with D-limonene, T0070907 or A939572 produced an additive reduction in lipid droplets, and these combinations were significantly more effective than either agent alone (Figures 9A–D). Conversely, D-limonene substantially attenuated the lipid deposition induced by rosiglitazone (Figures 9A–D). These results suggest that the protective effect of D-limonene against FFA-induced lipid accumulation depends at least in part on the suppression of PPARγ and its downstream effector SCD-1, functionally confirming the PPARγ/SCD-1 axis as the critical site of its therapeutic action.

To further verify the involvement of the PPARγ/SCD-1 axis at the molecular level, we performed Western blot analysis. As shown in Figure 9E, compared with the control group, FFA treatment significantly upregulated the protein expression of PPARγ and its downstream target SCD-1. Co-treatment with D-limonene markedly reduced the protein levels of both PPARγ and SCD-1, consistent with its lipid-lowering effect. The PPARγ antagonist T0070907 decreased PPARγ protein expression, whereas the SCD-1 inhibitor A939572 predominantly inhibited SCD-1 (Figures 9E–G). As expected, the PPARγ agonist rosiglitazone further increased PPARγ expression. Moreover, the combination of D-limonene with either T0070907 or A939572 resulted in greater suppression of PPARγ and SCD-1 than each agent alone, indicating an additive effect at the protein level (Figures 9F,G). These Western blot results provide convincing molecular evidence that D-limonene reduces FFA-induced lipid accumulation through the PPARγ/SCD-1 axis.

## 4 Discussion

Metabolic dysfunction-associated steatotic liver disease (MASLD) is a complex metabolic disorder affecting approximately 30% of the global adult population (Rinella et al., 2023; Younossi et al., 2016). It is clinically characterized by lipid dysregulation, oxidative stress, and chronic inflammation. Its pathogenesis involves intricate crosstalk among multiple pathways, and effective pharmacotherapies remain limited. Natural products, with their multi-target properties, favorable safety profiles, and diverse bioactivities, represent promising candidates for intervention. In this study, we employed an integrated strategy combining network analysis, transcriptomics, metabolomics, and experimental validation to systematically investigate the therapeutic effects and mechanism of the citrus-derived monoterpene D-limonene in MASLD. We suggest that D-limonene, at the tested doses, significantly alleviates hepatic steatosis, inflammatory infiltration, and liver injury, while reducing key pro-inflammatory mediators and restoring oxidative balance. Our experimental data suggest that D-limonene exerts its protective effect at least in part by modulating the PPARγ/SCD-1 axis and potentially affecting downstream lipid metabolic pathways.

A central feature of MASLD is hepatic lipid metabolic imbalance, driven by increased *de novo* lipogenesis, impaired fatty-acid oxidation, and reduced lipid export, ultimately leading to lipotoxicity, insulin resistance, and inflammation (Horn and Tacke, 2024). Our findings show that D-limonene effectively reduces HFD-induced hepatic lipid accumulation, which was confirmed by Oil Red O staining, and systemically improves the serum lipid profile by lowering TG, TC, LDL-C, and FFA while increasing HDL-C. These results are consistent with previous reports showing that D-limonene reduces serum and hepatic lipids in HFD-induced obese mice (Jing et al., 2013) and rats (Liao et al., 2023). However, the present study is the first to systematically evaluate its effects on the full lipid profile (including FFA and HDL-C) in a MASLD model, underscoring its potential to correct both systemic and intrahepatic lipid disturbances under the present experimental conditions.

To systematically elucidate the underlying mechanism, we integrated network analysis, transcriptomics, and metabolomics. Our unbiased multi-omics analysis consistently identified the PPAR signaling pathway as a candidate pathway potentially associated with D-limonene intervention (Ou et al., 2026). It is critical to emphasize that network analysis functions as a hypothesis-generating tool rather than a definitive mechanistic determinant; therefore, the PPAR pathway was prioritized for subsequent experimental validation based on its enrichment significance and biological relevance. While the PPAR family includes three subtypes (α, β, and γ), our integrated data specifically highlighted the modulation of the PPARγ-mediated lipogenic axis (Qiu et al., 2025). RT-qPCR validation further showed that D-limonene downregulated all three PPAR subtypes in the MASLD model. However, PPARα and PPARβ are known to promote lipid clearance when activated (Mastoridou et al., 2026); their paradoxical elevation in the disease state and suppression by D-limonene suggest they are compensatory responses rather than primary therapeutic targets. In contrast, PPARγ, a core driver of *de novo* lipogenesis and steatosis (Li et al., 2025), showed expression changes that directly paralleled disease progression and therapeutic outcomes. Therefore, we focused on the PPARγ/SCD1 signaling node for in-depth investigation. Our findings suggest that D-limonene effectively remodels hepatic lipid metabolism, particularly by targeting the PPARγ/SCD1 axis, thereby inhibiting excessive lipid accumulation in the context of the present single-dose paradigm.

Guided by this multi-omics evidence, we focused on PPARγ and its key downstream effector, SCD-1. SCD-1 catalyzes the rate-limiting step in monounsaturated fatty acid production and is transcriptionally activated by PPARγ(Yao-Borengasser et al., 2008). Their co-overexpression is a recognized driver of hepatic steatosis. Genetic deletion or pharmacological inhibition of SCD-1 protects against hepatic steatosis in rodent models (Miyazaki et al., 2007). Here, we show that D-limonene significantly downregulates PPARγ and SCD-1 at the mRNA and protein levels. Molecular dynamics simulations further support stable binding of D-limonene to both targets, with minimal conformational fluctuation over 100 ns. Functional validation using pharmacological modulators showed that the lipid-lowering effect of D-limonene was enhanced by the PPARγ antagonist T0070907 or the SCD-1 inhibitor A939572, and was counteracted by the PPARγ agonist rosiglitazone. These rescue experiments provide supportive functional evidence that the PPARγ/SCD-1 axis represents a key pharmacological node contributing the observed lipid-lowering effects of D-limonene under the present experimental conditions (Zhengdong et al., 2024). Our findings provide preliminary insights into the anti-steatotic actions of dietary natural products suggesting that D-limonene may dually modulate the PPARγ/SCD-1 axis.

As a dietary monoterpene abundant in citrus fruits, D-limonene holds unique development potential owing to its dietary and medicinal properties. Its long history of human exposure provides a strong safety foundation, markedly reducing pre-clinical and clinical toxicological risk compared to novel synthetic compounds. Our earlier dose selection safety considerations (National Toxicology Program, 1990; Ramos et al., 2015) further support that the doses used in this study (100 mg/kg and 300 mg/kg) are well within a safe window. However, toxicological safety and pharmacological plausibility are not equivalent concepts. The administered doses substantially exceed the estimated pharmacologically active threshold for pure d-limonene (<10 mg/kg, assuming <1% purity of the active metabolite), which necessarily constrains the translational inference of the observed effects. Moreover, its dual mechanism—simultaneously inhibiting PPARγ transcriptional activity and SCD-1 enzymatic function—aligns with modern polypharmacology strategies for complex metabolic diseases, potentially offering synergistic efficacy and reducing the risk of compensatory resistance seen with single-target therapies (Yang et al., 2025). Nevertheless, several challenges remain. Oral bioavailability and hepatic targeting may require further optimization through advanced formulation strategies such as nano-delivery systems. While the PPARγ/SCD-1 axis is identified as a candidate mechanism based on our integrated network analysis and experimental validation, D-limonene’s potential effects on the hepatic immune microenvironment and gut microbiota, which are both implicated in MASLD pathogenesis, warrant further investigation to fully map its therapeutic network.

Despite these findings, several limitations of this study warrant consideration. First, from an ethnopharmacological perspective, the dose of a pure compound derived from a botanical source should ideally be translatable from its estimated natural content (Heinrich et al., 2018). Based on literature reports, the content of D-limonene in dried citrus peels typically ranges from 0.95% to 3.56% (w/w) (Lopresto et al., 2014; Visvalingam et al., 2026). Assuming a traditional botanical dose of 1 g/kg, the corresponding ethnopharmacologically predicted dose of pure D-limonene would range from approximately 9.5 mg/kg to 36 mg/kg. Our employed dose (∼300 mg/kg) is roughly 8- to 30-fold higher than this predicted range, which substantially limits the translational relevance of our findings to traditional use. Second, although two doses (100 and 300 mg/kg) were tested, the dose-response relationship remains incompletely characterized due to the limited dose levels and the absence of a full range of lower concentrations. Moreover, the current dosing regimen (oral gavage and duration) may not be optimal and requires further refinement. Critically, whether the observed hepatoprotective effects can be replicated at pharmacologically relevant lower doses (e.g., 10–50 mg/kg) remains unknown. Accordingly, future studies should include systematic dose-response experiments with lower, physiologically justified concentrations, optimized administration protocols, and pharmacokinetic profiling.

It is also noteworthy that the combination of a suprapharmacological dose (from an ethnopharmacological perspective) and overinterpretation of *in silico* predictions is a recognized concern in natural product research (Panossian, 2025). The present study includes CETSA experiments that provide direct experimental evidence of D-limonene binding to PPARγ and SCD-1. However, network analysis results remain hypothesis-generating and do not demonstrate causality. Future studies employing surface plasmon resonance (SPR) or other direct binding kinetics assays are needed to further validate and quantify these interactions, and lower, pharmacologically relevant doses must be tested to confirm the therapeutic potential.

## 5 Conclusion

In summary, using integrated multi-omics and experimental validation, this study provides preliminary evidence that D-limonene, at the dose tested (300 mg/kg), ameliorates MASLD at least in part through an association with the PPARγ/SCD-1 signaling axis. The combined data suggest that D-limonene may suppress lipogenic gene transcription and reduce monounsaturated fatty acid production, thereby attenuating hepatic lipid accumulation and lipotoxicity. However, it is important to note that the dose used in this study is substantially higher than the dose predicted from an ethnopharmacological perspective, and future studies using lower, pharmacologically relevant doses, along with direct binding assays such as SPR, are necessary to validate these findings and to establish a definitive mechanism. Nevertheless, our results generate a testable hypothesis for further investigation into D-limonene as a potential lead compound for MASLD.

## Data Availability

The transcriptomics data presented in the study are deposited in the NCBI SRA repository, accession number PRJNA1478036. The metabolomics data presented in the study are deposited in the OMIX database of the China National Center for Bioinformation, accession number OMIX017892.
