# Supplementary material for: D-limonene ameliorates metabolic dysfunction-associated steatotic liver disease by inhibiting the PPARγ/SCD-1 pathway and improving lipid metabolism disorders
Source: Front Pharmacol. 2026 Jul 2;17:1843336. doi: 10.3389/fphar.2026.1843336 (PMC13372976; doi:10.3389/fphar.2026.1843336)
Supplement: Supplementary file 1 [file Supplementaryfile1.docx]

**Supporting Information**

**D-limonene ameliorates metabolic dysfunction-associated steatotic liver disease by inhibiting the PPARγ/SCD‑1 pathway and improving lipid metabolism disorders**

| **Genes** | **Forward primers (5’-3’)** | **Reverse primers (5’-3’)** |
| --- | --- | --- |
| *PPARγ* | \| ATGCACTGCCTATGAGCACTT \| \| --- \| | TGCTGGAGAAATCAACCGTG |
| *SCD-1* | CCTGTTCGTCAGCACCTTCT | TAGGGGAAGGCGTGATGGTA |
| *FABP4* | ACTTGGTCGTCATCCGGTCAG | GCTCTTCACTTTCCTGTCATCTGG |
| *FABP1* | GGAAGGACATCAAGGGGGTG | GTCATGGTCTCCAGTTCGCA |
| *PPARα* | CGATGCTGTCCTCCTTGATGAAC | GATGTCGCAGAATGGCTTCCTC |
| *PPARβ* | CCGACAGCCAGTACCTCTTCC | TCTCACTCTCCGTCTTCTTCAGC |

**Table S1** **Primers for RT-qPCR analysis**


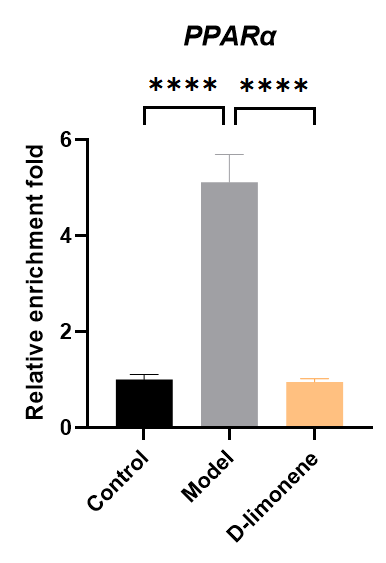

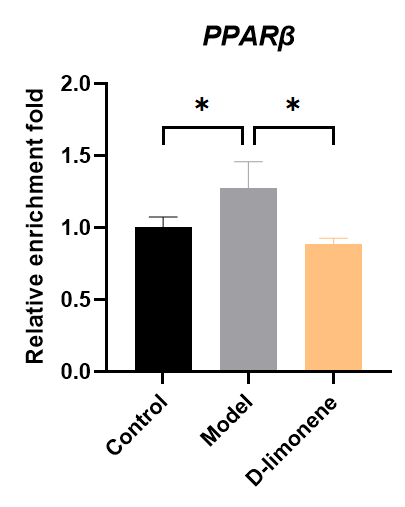


**Fig. S1 The mRNA expression levels of PPARα and PPARβ**
